# A New Automatic System for Angular Measurement and Calibration in Radiometric Instruments

**DOI:** 10.3390/s100403703

**Published:** 2010-04-13

**Authors:** Jose Manuel Andujar Marquez, Miguel Ángel Martínez Bohórquez, Jonathan Medina Garcia, Francisco Jose Aguilar Nieto

**Affiliations:** Departamento de Ingeniería Electrónica, de Sistemas Informáticos y Automática, Escuela Politécnica Superior de la Universidad de Huelva, Ctra. Palos de la Ftra.-Huelva s/n, 21819 Palos de la Frontera, Huelva, Spain; E-Mails: andujar@uhu.es (J.M.A.M.); jonathan.medina@diesia.uhu.es (J.M.G.); franciscojose.aguilar@alu.uhu.es (F.J.A.N.)

**Keywords:** angular response, calibration, cosine error, data acquisition, radiometric instrument, solar irradiance

## Abstract

This paper puts forward the design, construction and testing of a new automatic system for angular-response measurement and calibration in radiometric instruments. Its main characteristics include precision, speed, resolution, noise immunity, easy programming and operation. The developed system calculates the cosine error of the radiometer under test by means of a virtual instrument, from the measures it takes and through a mathematical procedure, thus allowing correcting the radiometer with the aim of preventing cosine error in its measurements.

## 1. Introduction

This paper puts forward the design, construction and testing of a new automatic system for angular-response measurement and calibration in radiometric instruments. Solar irradiance (energy falling on a surface measure, W/m^2^) measurement is mainly performed by radiometers [1]. The most widely-used radiometers are those measuring within the visible (radiation) [2] and ultraviolet (UV) spectra. For solar irradiance measurement, the radiometer’s input optics must provide appropriate angular response, also known as cosine [3,4] or Lambert-Law response. The Lambert Law (or Lambert’s cosine law) states that the lighting produced on a given surface by a punctual light source is proportional to the cosine of the angle of incidence. According to it, radiometer maximum response is obtained when solar irradiance falls perpendicularly on the radiometer sensor surface (the sun is at its zenith), while its minimum response is obtained when the sun is on the horizon (angle of incidence = 90°). For any other angles, instrument response follows the cosine function. Thus, for instance, for an incidence angle of 60°, the response is half of the maximum. The irradiance (*E*) measured by an ideal radiometer for any given solar zenith angle *θ* (angle to the perpendicular to the ground plane) is the product of the irradiance measured in the vertical *E_0_* by the cosine of that angle. Thus:

(1) $$E=E_{0}\;\text{cos}\;\theta$$

The angular response of real instruments deviates from the previous equation. In fact, most of them tend to underestimate actual solar irradiance. This fact is known as cosine error [5,6]. The cosine error of a given instrument is defined as the deviation between the actual angular response provided by the equipment and the ideal angular response [7] given by Equation (1).

Cosine error is one of the main causes of error in radiometric-instrument measurement [8] and its magnitude ranges from units to some percentage tens, depending on the angle of incidence [9], which hinders the comparison of data provided by instruments in different locations [10], hence the importance of intercomparisons of radiometric instruments in standardized labs at international level.

Radiometers measure global irradiance (*i.e.*, the sum of direct and diffuse irradiance (diffuse irradiance is produced by the dispersion effects of atmospheric components, including clouds), both of which present cosine error when measured). Several methods were designed for cosine-error analysis in radiometer measurement such as the measurement of direct and global, or diffuse and global irradiance, either at the same time or one immediately after another, as long as steady atmospheric conditions are kept along measurement. In general, we only dispose of global irradiance measures obtained by the equipment under test. The methodology used in this work is put forward in [8,11].

Reference [12] presents a different method for cosine-error measurement in a radiometric instrument by means of measurements from two broadband instruments aimed at studying the angular response of a Brewer spectroradiometer: one for global and the other for diffuse irradiance measurement. The works presented in [13,14] shall also be highlighted.

Instrument angular response mainly affects the direct component of global radiation. To a lower extent, diffuse radiation is mainly affected by two factors: (1) the diffuse radiation generated in close-to-horizon angles is a little part of total measured diffuse radiation, and (2) within the UV region, this radiation is even less as a result of the increasing ozone absorption due to the higher optical path length. In most broadband radiometers and spectroradiometers, the cosine error increases as the solar zenith angle does [15,16].

The cosine error may be reduced by means of different physical and/or mechanic procedures. Therefore, accurate knowledge of the instrument’s angular response becomes necessary, since it allows analytical instrument characterization [17]. To determine the angular response of a given radiometric instrument, different trials and tests must be completed both in lab and in the study field [18,19].

The angular response in radiometric instruments is currently characterized manually. The system presented here is an automatic instrument to measure and calibrate radiometric instruments with no human handling. The developed system has been operating since September 2007 in the lab of radiometric-instrument test and characterization in the INTA (Spanish National Institute of Aerospace Technique), in its headquarters in El Arenosillo (Mazagón, Huelva, Spain). This system was successfully tested first in an international campaign on intercomparison in 2007 for the measurement and calibration of 22 radiometric instruments from different countries [20]. The results obtained with this system are much better than those previously obtained through manual trials. The developed equipment improves measurement reliability, resolution and precision, and reduces the time necessary for angular response determination. Moreover, it also leads to noticeable improvement regarding its capacity to immunize experiments against electromagnetic and light noise, since tests are performed in a dark chamber with no human presence or handling. The developed system is registered at the Spanish Patent and Trademark Office under the reference P200703162, PCT/ES2009/000042 and international reference WO/2009/095515.

The present paper is organized as follows: Section 2 describes the system’s modules and elements. Section 3 explains system-testing aimed at assuring design requirements are met regarding both accuracy and precision. Section 4 describes the operational process for radiometer measurement and calibration by means of the developed system. The conclusions of this work are contributed in Section 5. Finally, this paper also includes an appendix and an acknowledgment section.

## 2. System Description

**Figure 1.**
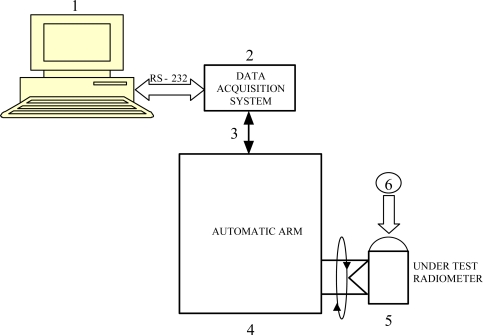
General block diagram for the developed system.

The block diagram of this system for angular-response measurement and calibration in radiometric instruments is shown in Figure 1. This figure only intends to illustrate the main system components, since its mechanical operation and movement capacity is illustrated in subsequent figures.

**Figure 2.**
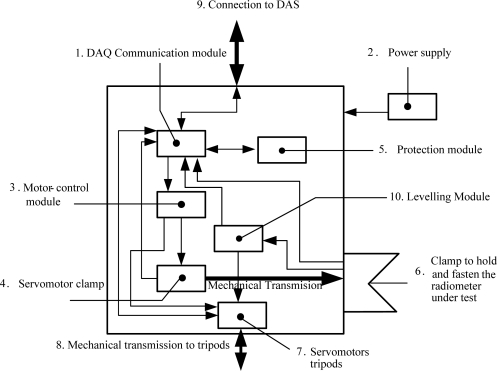
Block diagram of the automatic arm.

The system is formed by an automatic arm [Figure 1(4)] (it is known as a “automatic arm”, since it includes a clamp-shaped device which holds and moves the radiometer under test [Figure 1(5)] with great precision, although it bears no resemblance to the shape of a human arm). During the test, the radiometer is lighted by a calibrated lamp [Figure 1(6)] whose radiation spectrum covers the radiometer’s measurement spectrum [21]. With the aim of subjecting the radiometer under test to the whole range of angles of solar incidence by means of the calibrated lamp, the automatic arm spins from +90° to −90° (angle to the vertical to the ground plane), thus obtaining and registering radiometer’s measurements for each angle, which are collected and processed by a high-performance Data Acquisition System (DAS) [Figure 1(2)] acting as an interface between the user and the PC. Digital Multimeter (DMM) Agilent™ 34970A [22] is used in this application for DAS (using a DMM of these characteristics as DAS may seem exaggerated. However, its high precision, easy operation and, mainly, its current availability are the reasons behind this choice). The precision of this DMM is 6½ digits, which includes a DAS-card with both analogical and digital inputs and outputs. 600 readings per second can be stored through each channel, with an exploration speed of up to 250 channels per second. Obtained signals are sent to a PC [Figure 1(1)] through RS-232 serial communication and subsequently stored and processed at the PC to obtain the necessary parameters allowing angular-error measurement and radiometer calibration. Overall system operation is also controlled through the PC. A virtual instrument (VI), programmed and designed in LabVIEW™, also controls the measure-acquisition and measure-processing systems. Batch-shield wires are used for DMM-automatic arm connections [Figure 1(3)] to avoid undesired-signal (noise) feedback, since voltage levels at radiometer output are of the order of mV. Undoubtedly, the most important subsystem is the automatic arm, whose block diagram is shown in Figure 2. Each of the blocks shall be described next.

### 2.1. DAS-automatic Arm Communication Module

**Figure 3.**
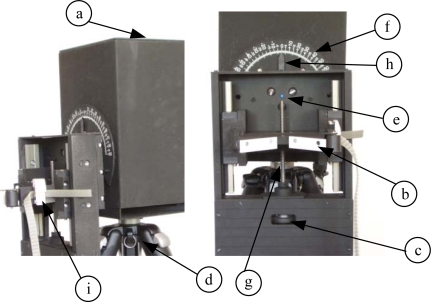
Front and profile view of the automatic arm.
Automatic arm main bodyClamps to hold the radiometer under testWheel for accurate levelling adjustmentElectronic tripod with levelling motorsScreeding LED indicator1°-step graduated scale (±90°) (for visual information only)Levelling worm gearPointerAnchorage for the radiometer under test fixation bridle.

The DAS communication module [Figure 2(1)] is a card gathering the automatic arm’s input and output signals coming from DAS. By means of the VI, the PC sends DAS the necessary commands for: (1) Placing the arm’s motors and tripod leveller [Figure 3(d)]. Set-point voltages for motor government are analogical signals of 0, 2.5 and 5 V reaching the controlling module of the arm’s motors [Figure 2(3)]. This module is responsible for generating the PWM control signal of all motors; (2) Turning on, by means of a digital output, the screeding light-emitting diode (LED) indicator [see Figure 3(e)]. This indicator reports alignment between the spinning centre of the automatic arm’s holding clamp and the radiometer’s sensor element; (3) Collecting the analogical voltage-output signal of each radiometer by means of an analogical voltage input. This signal (the irradiance measure of the radiometer under test) is digitalized, sent and processed at the PC; and, finally, (4) Receiving possible anomalies by means of digital inputs and monitoring automatic-arm operation signals. On the other hand, DAS sends the PC signals of (1) Output voltage and angular position of each radiometer under test; and (2) The automatic arm’s state-control signals. All these PC-automatic arm communication signals are transmitted by means of a 6-conductor, batch-shield multi-polar wire [Figure 2(10)].

### 2.2. Power Supply

The power supply [Figure 2(2)] provides the automatic system with the necessary energy to operate and power its motors and control electronics. It is connected as an external module not to influence measurements, since its transformer may induce noises (the power for the whole system is 220 AC). The power source is short-circuitable and capable of supplying up to 2 A, and disposes of a ±12 V-output.

### 2.3. Motor-control Module

The motor-control module [Figure 2(3)] comprises two identical commercial cards (MD22 [23]: Devantech Ltd.). Each of these cards can control two medium-power continuous-current motors. The servomotor placing the clamp which fastens and holds the radiometer under test is connected to one of the card outputs [Figure 2(4)]. The other three available card outputs control the tripod’s three levelling servomotors guaranteeing the radiometer under test is kept parallel to the ground for a zenith angle of 0°. Cards accept 5 different kinds of control. Precisely, the one with two analogical inputs is used here. The motor-control module works with a set-point signal of 0 V for the maximum speed in one spinning direction, 2.5 V for the central resting position and 5 V for the maximum spinning speed in the other direction. According to the received set-point signal (0, 2.5 or 5 V), the module generates the necessary PWM signal for motor control [Figure 2(4 and 7)]. Servomotors stop when: (1) The clamp has placed the radiometer in the desired angle, and (2) The system has placed the radiometer parallel to the ground.

### 2.4. Servomotor Clamp Module

**Figure 4.**
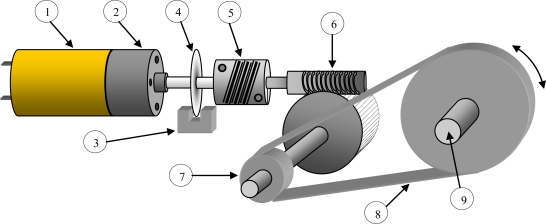
Servomotor arm module and mechanical transmission.

**Figure 5.**
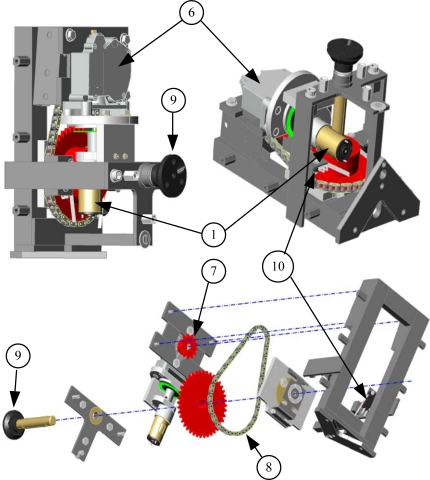
3D picture of the servomotor module and the clamp-moving transmission.

The servomotor module [Figure 2(4)] and clamp-moving transmission required special and detailed design, since radiometer measurement and calibration systems must be highly precise and reliable. Therefore, the system which places the automatic arm’s clamp holding the radiometer under test must fulfil a series of characteristics related to mechanical precision and reliability to assure high-precision measurements. With this aim, a mechanical coupling (see Figures 4 and 5) between the dragging motor and the axis holding the clamp-device was designed to guarantee steady transmission involves no mechanical fatigue, lifetime adjustment (no play and no maintenance) and 1/60-degree angular resolution. This resolution is much higher than that used so far for radiometer measurement and calibration. Tripod-moving servomotors [Figure 2(7)] are not particularly highlighted, as they are connected to each of the three legs of the tripod by a much simpler and less demanding coupling than that shown in Figures 4 and 5: a coupling to a worm drive which regulates the height of each tripod. The elements of the transmission system of the clamp servomotor (Figure 4) are described next:

1. **DC Servomotor**: The motor used is a continuous-current geared motor
2. **Gear reducer**: It is the first reduction stage and belongs to the short block
3. **Sensor and optic encoder barrier**: It is a standard kind of infrared-beam sensors/detectors: sensor TCST 1030 —located on both sides of the optical barrier—emits and receives light, which is interrupted by the perforated disk (4)
4. **Encoder perforated disk**: The disk used contains 120 perforations per revolution. However, encoder measurement (3) is made with double quadrature, so as to produce 240 pulses along a complete revolution. Therefore, resolution is 360/240 (1.5 degrees). However, as we shall see later on, this number of pulses is multiplied by the relation existing in the transmission up to the main axis (9), which pushes the system’s final resolution over 1.5 degrees
5. **Homokinetic coupling**: This aluminium coupling is inserted between the first (2) and second (6) reduction boxes, thus helping to avoid possible transmission mismatches and being highly-recommendable for precision couplings
6. **Helical worm-drive gear reducer**: This gear-reduction stage is based on a crown including a worm drive or helical gear assembly which allows blocking the final axis when the motor is not operating. Its adjustments show no play. The reduction relation of the helical gear assembly is 30:1, which means that the screw (6) spins once for every 30 motor-axis revolutions
7. **Chain pinion**: This is the last reduction relation. It is performed with chain-assembled steel pinions. Pinions keep a 3:1 ratio and are capable of standing much higher pairs of forces than those demanded by the automatic arm, thus avoiding possible fatigue risks
8. **Metallic chain**: It is used to assure no transmission displacement and avoid plays and maintenance. Its breakage stress is unreachable, thus offering lifetime guarantee
9. **Final positioning axis**: This axis is responsible for transmitting movement to the arm’s clamp system which holds and moves the radiometer under test. The stainless steel with special machining it is made out of allows leading the wires powering the levelling LED through its interior, thus avoiding them getting caught with the arm’s clamp movements
10. **Limit switch** (see Figure 5(10)): It is a protection in case of software failure. The motor stops when maximum 90° spinning is exceeded by the clamp holding the radiometer.

According to the encoder perforated disk and reduction steps (6–7), the resolution of the developed system is:

(2) $$\frac{360}{240}\cdot \frac{1}{30}\cdot \frac{1}{3}=\frac{360}{21600}=\frac{1}{60}\;\text{degrees}$$

This means that, for an encoder pulse (1.5 degrees in the motor axis), a 1/60-degree resolution is obtained in the clamp holding and positioning the radiometer.

Figure 5 shows a 3D picture of the servomotor module and the clamp-moving transmission. The numbers in this figure agree with those in Figure 4.

### 2.5. Protection Module

The protection module [Figure 2(5)] protects the device against failures. It includes a limit switch which prevents the clamp holding the radiometer from spinning more than 90°, and the fuses which protect system electronics. With the aim of avoiding the dependence of electronic components on power and temperature, constant-voltage sources were also used in circuits.

### 2.6. Clamp for Radiometer Holding and Moving

**Figure 6.**
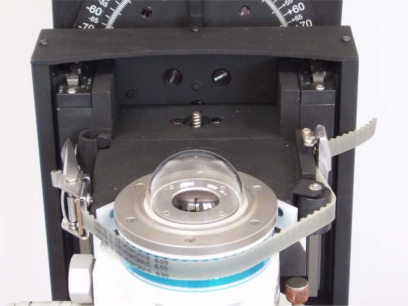
Detail of the system to fasten the radiometer under test on to the automatic arm.

The system which holds and moves the radiometer under test [Figure 2(6)] is clamp-shaped [Figure 3b]. To position the radiometer in each measurement angle, the clamp is moved by a servomotor (Figure 4) whose axis is coupled to a wheel for flush adjustment [Figure 3c]. This operation is performed manually (currently being automated for a new version of the developed system) prior to test onset. The radiometer is fastened with an adjustable strap (Figure 6), so it is always moved jointly with the fastening clamp.

### 2.7. The Levelling Module

Finally, the levelling module [Figure 2(10)] is aimed at keeping the radiometer parallel to the ground (when solar zenith angle *θ* = 0°). Measurements are taken in relation to the horizontal of the plane. Therefore, the system must be capable of levelling itself horizontally. Highly reliable position sensors are used with this purpose: accelerometers working as 2-axis inclinometers (ADXL 203, Analog Devices™) capable of determining automatic-arm inclination with high precision and whether levelling in relation to the horizontal is necessary. The accuracy of the levelling system is ±0.1°. The levelling module—apart from receiving the signals from the sensors and sending them to the VI—also governs the tripod’s servomotors automatically (see Figure 2).

## 3. Calibration and Testing

As a previous step to radiometric-instrument measurement and calibration, the developed system was tested to assure it fulfils the afore-mentioned precision, resolution and reliability requirements. Thus, a test was performed in lab by means of laser interferometer XL 80 (Renishaw™). The precision of this linear and angular measurement system is guaranteed by its manufacturer (±0.5 ppm). Readings can be obtained up to a maximum speed of 4 m/s with 1-nm resolution. An additional device (RX 10) is used for angular measurement, since its precision reaches up to 1 arc-second (±5 μm).

In the calibration process, an experiment was designed by means of VI programming, so as to make the automatic arm’s clamp cover all the angles between ±90° in 0.1°-steps. By the laser interferometer it was observed that error always remains below 0.01%. Next, it was ascertained that 1/60-degree angular resolution is reached. With this purpose, the VI was programmed to make the automatic arm’s clamp spin pulse by pulse the order of the optical encoder, bearing in mind that—according to its design—1.5 motor-axis pulses are equivalent to 1/60 degrees in the clamp. 60 measures were taken in each ±90° trial. 100 equal trials were completed and all measures ranged between 1/60 ± 10% degrees.

## 4. Field use of the System. Experimentation

The developed system has been operating since September 2007 in the lab for radiometric-instrument assay and characterization in the headquarters of the INTA (Spanish National Institute of Aerospace Technique) in El Arenosillo (Mazagón, Huelva, Spain). This system was successfully tested first in an international campaign on intercomparison in 2007 for the measurement and calibration of 22 radiometric instruments from different countries [20].

### 

#### Use of the Developed System in the Lab of Radiometric-instrument Test and Characterization

So far, this paper only deals with the measurement of the response of the radiometer under test to radiation received from different angles (±90°). However, for subsequent calibration, the mathematical procedure detailed in Appendix 1 must be applied for cosine-error calculation. The sum of both steps guarantees the radiometer’s reliability, since its cosine error for each angle is known.

The operation procedure with the developed system is as follows: The radiometer under test is placed into the arm’s clamp and fastened (Figure 6). Next, the VI is started. Firstly, the radiometer is levelled in relation to the ground plane (the former must be parallel to the latter). After levelling, the VI shall show the corresponding confirmation message. Subsequently, the user performs manual screeding (currently being automated for a new version of the system) of the radiometer under test (Figure 3c): vertical adjustment to align the rotation axis of the automatic arm with the sensor element of the radiometer. Next, the measurement-onset button is pressed and—after a user-adjustable time-period for leaving the test room—the measurement process begins with the values specified by the user. These values are: initial and final angular value (usually ±90°), value of each step (choice: 0.1°, 1°, 2°, *etc.*), and time for measure acquisition in each step. Once these data are provided, data collection begins according to the programmed path. Results are finally displayed in screen and saved into a file.

**Figure 7.**
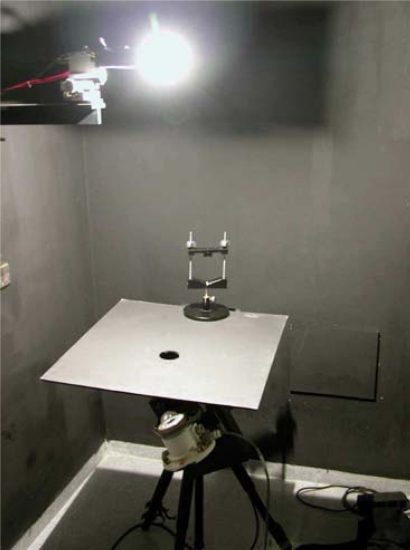
Radiometer under test photography.

Next, the usual operation procedure is described. The measuring procedure in lab consists of rotating the radiometer at a constant distance from a calibrated lamp (see Figure 7). This, together with the pierced screen placed between the radiometer and the lamp, allows considering the lamp filament as a point source. Figure 7 shows an radiometer moved by the automatic system developed in this work. It is very important to ensure the lamp is perpendicular to the radiometer sensor when the angle of incidence is zero above the rotating system, since—if this were not the case—false results may be provided at higher angles. An external laser is used to perform this alignment. The automated system carries out an initial measurement at 0° and starts to spin one degree at a time to take measurements from −90° to +90°.

**Figure 8.**
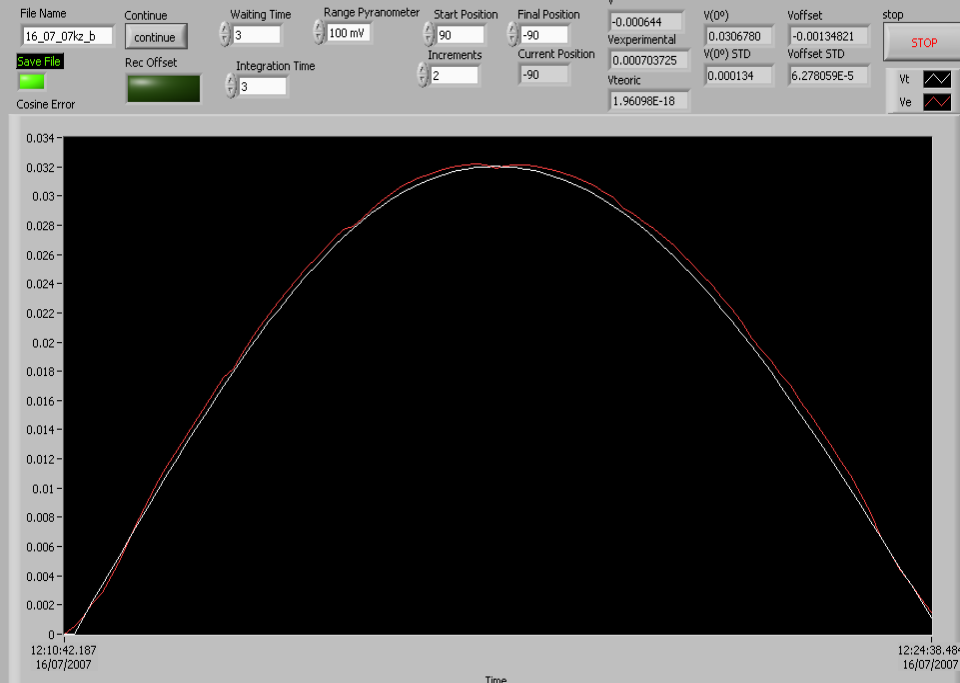
Ideal response and real measured response curves for a given radiometer according to the angle of incidence.

Figure 8 shows the measures taken by the developed system for a given radiometer. The VI graphs and displays these measures (the screenshot in this case is the VI’s *cosine-error* section, since it governs the whole system and the measure-taking and measure-processing processes). The curves shown in Figure 8 represent the ideal output of the radiometer under test (cosine response) in white and its real output in red, both according to the radiation’s angle of incidence from the vertical. The vertical axis is graduated in volts and the horizontal axis shows test time. Since the radiometer’s ideal response must be analogous to the cosine of the radiation’s angle of incidence, its response must be maximum (100%) when radiation is perpendicular to the sensor surface (sun zenith). Its response must be 50% when the angle of incidence is 60° and 0% when the sun is on the horizon (90°).

Following the described procedure, the developed system measured and calibrated the radiometric instruments in the international campaign on intercomparison which took place in the INTA facilities in El Arenosillo (Huelva, Spain) between 15 August and 21 September 2007. These international campaigns are usually organized every one or two years in different standardized scientific centres worldwide, following World Meteorological Organization (WMO) recommendations [24]. Obtained results were excellent and are reported in [20].

## 5. Conclusions

This paper puts forward the design, construction and testing of an automatic system for angular-response measurement and calibration in radiometric instruments. This system includes an automatic arm which, by means of its clamp, automatically holds and places the radiometer under test to cover, in programmable steps (degrees and tenths of degree), the movement of solar irradiance falling onto a radiometer in normal operation (±90°). Radiometer test is performed with a calibrated lamp as a radiation source whose radiation spectrum covers the radiometer’s measurement spectrum. The system also includes a high-performance DAS and a PC as a controller by means of a specifically-designed VI.

The developed system allows much higher resolution than that reached so far, since 1/60 degrees is reached for all angles of incidence. This is achieved by means of the mechanical transmission system located in the arm’s clamp and the incorporated servomotor. To guarantee the perpendicularity of the radiometer under test when the light source is at its zenith (solar zenith angle = 0°), the developed system automatically levels itself.

Regarding the calibration of tested instruments, the VI calculates—from the given measures and by means of the mathematical procedure in Appendix 1—the cosine error of the instrument, which allows correcting the tested radiometer in its current location, thus eliminating cosine error from its measurements. The system developed in this work has been operating successfully, with no breakdowns, since September 2007 in the lab of radiometric-instrument test and characterization in the headquarters of the INTA (Spanish National Institute of Aerospace Technique) in El Arenosillo (Mazagón, Huelva, Spain). Until the arrival of this system to lab, the process of radiometer measurement and calibration was performed manually, with much less measurement precision, resolution and quality, thus also being much more time-consuming. Apart from the foregoing, the avoidance of errors due to human handling must also be taken into account.

The system developed in this work was registered at the Spanish Patent and Trademark Office under reference P200703162, PCT/ES2009/000042 and international reference WO/2009/095515.

## Appendix: cosine-error calculation in radiometric instruments

There are several methods used to calculate the value of the cosine error in radiometric devices. Some manufacturers use simplified expressions such as those in [25]. Nevertheless, the procedure explained next is widely used in research and calibration centres.

Radiometer-measured irradiance should follow the ideal angular response given by the cosine law. Deviations from this angular response produce systematic measurement errors which do not only depend on solar zenith angle but also on the division of solar radiation into direct and diffuse [26]. The cosine correction function used to correct these deviations is based on the following expression:

(A.1) $$\mathrm{COSCOR}(\theta )=\frac{1}{f_{b}(\theta )\cdot r(\theta )+f_{d}\cdot (1-r(\theta ))}$$

where *f_b_*(*θ*) and *f_d_* stand for the correction factors for direct and diffuse solar radiation, respectively, and *r*(*θ*) is the ratio between the direct and the global effective irradiance. Thus, *r*(*θ*) and 1−*r*(*θ*) stand for the proportions of the direct and diffuse components composing global irradiance.

The correction factor for direct irradiance can be calculated from measurements performed in lab and defined as:

(A.2) $$f_{b}(\theta )=\frac{U}{U(\theta =0)\cdot \;\text{cos}\;\theta },$$

where *U* is the voltage signal measured for zenith angle and *θ* is the angle in relation to zenith.

The correction factor for diffuse irradiance can be calculated from the previously-calculated correction factor for direct irradiance by the following expression [27]:

(A.3) $$f_{d}=2\int_{0}^{\pi /2}f_{b}(\theta )\;\text{sin}\;\theta \;\text{cos}\;\theta \;d\theta$$

In order to apply this correction, it is necessary to assume an angular response error independent from the azimuth angle (*θ*) and an isotropic diffuse solar radiation field. The first assumption is acceptable for UV radiation since—for solar zenith angles below 80°—changes in azimuth angle produce variations in angular response errors within ±1% [28].

The last missing term to complete Equation (A.1) is the ratio between direct and global effective irradiance *r*(*θ*), which is calculated by the following expression:

(A.4) $$r(\theta )=\frac{\int_{280\mathrm{nm}}^{400\mathrm{nm}}\mathrm{SRF}(\lambda )E_{b}(\theta ,300\mathrm{DU},\lambda )d\lambda }{\int_{280\mathrm{nm}}^{400\mathrm{nm}}\mathrm{SRF}(\lambda )E_{g}(\theta ,300\mathrm{DU},\lambda )d\lambda }$$

where *SRF(λ)* is the radiometer’s relative spectral function, and *E_b_*(*θ*,300*DU*,*λ*) and *E_g_*(*θ*,300*DU*,*λ*) stand for spectral direct and global irradiance, respectively, as calculated by a radiative transfer model considering a constant total ozone column of 300 DU (Dobson Units). Note that the direct/global effective irradiance ratio must be computed for each broadband radiometer, since its calculation involves the actual spectral response of each instrument.

Once Equations A.2, A.3 and A.4 are computed, the cosine correction function (A.1) for any given under test radiometer is immediately calculated.
